# A comprehensive analysis of biomarkers associated with synovitis and chondrocyte apoptosis in osteoarthritis

**DOI:** 10.3389/fimmu.2023.1149686

**Published:** 2023-07-21

**Authors:** Ling Yang, Xueyuan Yu, Meng Liu, Yang Cao

**Affiliations:** ^1^Department of Hematology, The First People’s Hospital of Changzhou, Third Affiliated Hospital of Soochow University, Changzhou, China; ^2^Department of Traditional Chinese Medicine, Xinhua Hospital Affiliated to Shanghai Jiao Tong University School of Medicine, Shanghai, China; ^3^Department of Plastic, Aesthetic and Maxillofacial Surgery, The First Affiliated Hospital of Xi’an Jiao Tong University, Xi’an, China; ^4^Department of Clinical Laboratory,The First Affiliated Hospital of Xi’an Jiao Tong University, Xi’an, China

**Keywords:** osteoarthritis, synovitis, cartilage apoptosis, immune infiltration, bioinformatics analysis

## Abstract

**Introduction:**

Osteoarthritis (OA) is a chronic disease with high morbidity and disability rates whose molecular mechanism remains unclear. This study sought to identify OA markers associated with synovitis and cartilage apoptosis by bioinformatics analysis.

**Methods:**

A total of five gene-expression profiles were selected from the Gene Expression Omnibus database. We combined the GEO with the GeneCards database and performed Gene Ontology and Kyoto Encyclopedia of Genes and Genome analyses; then, the least absolute shrinkage and selection operator (LASSO) algorithm was used to identify the characteristic genes, and a predictive risk score was established. We used the uniform manifold approximation and projection (UMAP) method to identify subtypes of OA patients, while the CytoHubba algorithm and GOSemSim R package were used to screen out hub genes. Next, an immunological assessment was performed using single-sample gene set enrichment analysis and CIBERSORTx.

**Results:**

A total of 56OA-related differential genes were selected, and 10 characteristic genes were identified by the LASSO algorithm. OA samples were classified into cluster 1 and cluster 2 subtypes byUMAP, and the clustering results showed that the characteristic genes were significantly different between these groups. MYOC, CYP4B1, P2RY14, ADIPOQ, PLIN1, MFAP5, and LYVE1 were highly expressed in cluster 2, and ANKHLRC15, CEMIP, GPR88, CSN1S1, TAC1, and SPP1 were highly expressed in cluster 1. Protein–protein interaction network analysis showed that MMP9, COL1A, and IGF1 were high nodes, and the differential genes affected the IL-17 pathway and tumor necrosis factor pathway. The GOSemSim R package showed that ADIPOQ, COL1A, and SPP1 are closely related to the function of 31 hub genes. In addition, it was determined that mmp9 and Fos interact with multiple transcription factors, and the ssGSEA and CIBERSORTx algorithms revealed significant differences in immune infiltration between the two OA subtypes. Finally, a qPCR experiment was performed to explore the important genes in rat cartilage and synovium tissues; the qPCR results showed that COL1A and IL-17A were both highly expressed in synovitis tissues and cartilage tissues of OA rats, which is consistent with the predicted results.

**Discussion:**

In the future, common therapeutic targets might be found forsimultaneous remissions of both phenotypes of OA.

## 1 Introduction

Osteoarthritis (OA), the most common form of arthritis, is characterized by chronic pain and high incidence (1) and disability (2) rates. OA arises from a complex process involving the cartilage, bone, synovium, ligaments, infrapatellar fat pads, meniscus, and muscles (3). Among them, the representatives that are most often studied are two significantly altered hallmarks, cartilage apoptosis and synovitis, whose discovery has often been considered a breakthrough in research on optimal treatment strategies for OA. Synovial inflammation usually occurs in the early stage of OA. Synovial inflammation can lead to the infiltration of inflammatory cells and the release of inflammatory factors, which can lead to cartilage destruction and joint dysfunction (4). However, unambiguous therapeutic targets and the correlation between the two phenotypes remain to be discovered, and we hoped in this research to identify genes or pathways significantly related to both synovitis and cartilage apoptosis to further interrogate the mechanism and effective therapeutic targets.

Clinical basic and systems biology studies have been performed to detect the pathogenesis of OA (5). Many OA-related protein markers or pathways play a role in the development and progression of OA, including endoplasmic reticulum, stress marker glucose‐regulated protein 78 (*GRP78*), and Bcl2‐associated athanogene 1 (*bag1*) (6). Transient receptor potential vanilloid 1 (*TRPV1*) is closely related to pain perception by OA patients (7). What is more? The roles of disintegrin and metalloproteinase with thrombospondin motif 5 (*ADAMTS5*) and follistatin-like protein 1 (*FSTL1*) in OA diagnosis and prognosis (8) have been reported. As for pathways, the Ca^2+^/CaMKII/Nrf2 signaling pathway could inhibit M1 macrophage polarization to attenuate synovium in OA (7), and a promotional effect of the *JUNB*/*FBXO21*/*ERK* axis on cartilage degeneration in osteoarthritis by autophagy inhibition (9) was also reported. However, the studies above only explored the mechanism or potential target from the perspective of a single phenotype, and their sample numbers were limited. A systematic high-throughput analysis of targets and pathways associated with two or more phenotypes of OA is needed.

Some systematic bioinformatic analyses have partly improved on the defects above. The *FoxO* and *IL-17* signaling pathways are likely to regulate OA progression according to Kyoto Encyclopedia of Genes and Genomes (KEGG) enrichment, and ubiquitylation was found to be a key bioactive reaction in OA after analyzing the molecular function and protein–protein interaction (PPI) results (9). Abnormally methylated differentially expressed genes (DEGs) in OA such as *COL3A1*, *LUM*, and *MMP2* are potential methylation biomarkers of OA, and *THBS2* might play a role in the end stage of the disease (10). However, these studies all have defects as they lack multi-omics analyses and are pending multi-dimensional validation. Recently, a bioinformatics-led investigation used the Gene Ontology (GO) and KEGG databases, the CIBERSORTx method, and the ConsensusClusterPlus R package to perform enrichment and immune infiltration analyses before ultimately differentiating immunity patterns into two clusters and validating the expressions of *TCA1*, *TLR7*, *MMP9*, *CXCL10*, *CXCL13*, *HLA-DRA*, *ADIPOQ*, and *SPP1* using qPCR in chondrocytes (5). However, a synovitis analysis was not performed in this comprehensive and systematic research. Therefore, a systematic, multi-dimensional analysis covering multiple phenotypes should be performed.

In this study, we combined genes from the Gene Expression Omnibus and GeneCards databases to find OA-related genes, then constructed a risk model and used the receiver operating characteristic (ROC) curve to screen out and evaluate 10 characteristic genes. Network analysis and functional analysis of two subtypes were performed to estimate the degrees of immune infiltration, and the results were finally validated by qRT-PCR in rats’ tissues.

## 2 Methods

### 2.1 Data download

We first downloaded the following five datasets associated with osteoarthritis from the Gene Expression Omnibus (GEO) database: GSE55457 (11), GSE12021 (GPL96) (12), GSE55235 (11), GSE12021 (GPL97) (12), and GSE82107 (13). Among these, the GSE55457, GSE12021 (GPL96), and GSE55235 datasets were used as osteoarthritis diagnostic model training sets, whereas the GSE12021 (GPL97) and GSE82107 datasets were used as osteoarthritis diagnostic model validation sets.

The osteoarthritis diagnostic model training sets were created by extracting and merging a common expression profile from GSE55457, GSE12021 (GPL96), and GSE55235, which contain 10 osteoarthritic synovial tissue samples and 10 control synovial tissue samples, 10 osteoarthritic synovial tissue samples and 9 control synovial tissue samples, and 10 osteoarthritic synovial tissue samples and 10 control synovial tissue samples, respectively. We used the “Combat” function in the sva R package (14) to correct a batch effect of merged data of 30 osteoarthritic synovial tissue samples and 29 control synovial tissue samples. The distribution of target gene-expression levels before and after the correction was visualized by box plot.

GSE12021 contains 10 osteoarthritic synovial tissue samples and 4 control synovial tissue samples, whereas GSE82107 contains 10 and 7 samples, respectively. All the samples are of human origin, and all the datasets are from the GPL97 platform (**Table 1**).

**Table 1:** The datasets are from the GEO database.

GSE	GPL	Species	Tissue Source	OA sample number	Control sample number
GSE55457	GPL96	Homo sapiens	Synovium	10	10
GSE12021	GPL96	Homo sapiens	Synovium	10	9
GSE55235	GPL96	Homo sapiens	Synovium	10	10
GSE12021	GPL97	Homo sapiens	Synovium	10	4
GSE82107	GPL570	Homo sapiens	Synovium	10	7

### 2.2 Identification of OA DEGs

We input the keywords “synovitis” and “chondrocyte apoptosis” into the GeneCards database to obtain synovitis-related and chondrocyte apoptosis-related genes (15) (**Supplementary Table 1**). Then, we defined osteoarthritis-related genes by taking the intersection of synovitis-related genes, chondrocyte apoptosis-related genes, and osteoarthritis diagnostic model training sets. The results are shown using Venn diagrams.

To estimate the impact of osteoarthritis-related gene-expression levels on the severity of osteoarthritis, differential gene-expression analysis of OA and control samples of integrated datasets was performed using the limma R package (16). A differential gene was defined by a threshold of |fold change (FC)| > 1.5 and *p*< 0.05; genes with FC > 1.5 and *p*< 0.05 were considered up-regulated genes and those with FC< -1.5 and *p*< 0.05 were considered down-regulated genes. We took the intersection of differential genes and x1-related genes and obtained differentially expressed osteoarthritis-related genes. The results are visualized using volcano plots.

### 2.3 Constructing a forest model and nomogram model

We used the least absolute shrinkage and selection operator (LASSO) analysis method to perform dimension reduction analysis and obtained the characteristic genes from differentially expressed osteoarthritis-related genes. For normalized gene-expression values of weighted coefficients penalty of the characteristic genes, we established a risk score formula and visualized them by forest maps.


$$\text{riskScore}=\;\sum_{\text{i}}\text{Coefficient }({\text{gene}}_{\text{i}})*\text{mRNA Expression }({\text{gene}}_{\text{i}})$$

A nomogram was constructed according to selected characteristic genes to forecast the prevalence of OA. Then, the model’s accuracy was tested using an independent validation dataset.

### 2.4 The molecular subtype of OA

Uniform manifold approximation and projection (UMAP), a non-linear dimensionality-reduction algorithm, was used to partition and compress a group of patients into clusters based on the given feature. Then, the characteristic genes provided the basis to identify these patients’ subtypes using the umap R package (17).

### 2.5 The assessment of biological characteristics among subtypes of OA patients

Gene function enrichment could be performed by GO enrichment analysis from different dimensions and levels, i.e., biological process, molecular function, and cellular component categories (18). The KEGG database extensively includes related genomes, biological pathways, drugs and diseases, and so on (19). We used the clusterProfiler R package (20, 21) to perform GO functional annotation and KEGG pathway enrichment to identify the significantly enriched biological processes of DEGs of different subtypes in OA patients, with the significance threshold of enrichment analysis set at *p*< 0.05.

Gene set enrichment analysis (GSEA) could confirm whether a group of pre-defined genes was statistically different between two biological states; this approach is commonly used to estimate a sample’s pathway and biological process activity (22). To analyze the differences in biological processes of different subtypes of OA patients, we downloaded “c5.go.v7.4.entrez.gmt” and “c2.cp.kegg.v7.4.entrez.gmt” based on gene-expression profile data (23). Then, GSEA was performed with the clusterProfiler R package to analyze enrichment and visualize the dataset.

Gene set variation analysis (GSVA) is a non-parametric unsupervised analysis method able to convert a gene’s expression matrix to a gene set’s expression matrix between different samples to estimate gene set enrichment in order to assess metabolic pathway enrichment among samples (24). To study the variation in biological processes among different subtypes, we used the GSVA R package (24) on account of the gene-expression profile of different samples of OA subtypes. The reference dataset “h.all.v7.4.symbols.gmt” was downloaded from the MSigDB database (23) to calculate a single sample’s enrichment score for each hallmark.

### 2.6 PPI analysis

There are universal inter-relationships between genes, especially between those able to regulate the same biological process. To reveal the connection between patients with different subtypes of OA, we constructed PPI networks on account of their DEGs. We obtained PPI data from STRING (25), using a score of 700 points as the threshold. After exporting PPI data, we conducted a further analysis using Cytoscape (Institute for Systems Biology, Seattle, WA, USA) (26), which contains the following 12 algorithms (27): Betweenness, BottleNeck, Closeness, ClusteringCoefficien, Degre, DMN, EcCentricity, EPC, MCC, MNC, Radiality, and Stress. We calculated the top 30 nodes in each algorithm and defined the “hub node” as the gene that appeared in at least five algorithms. Hub nodes have a greater level of connection with others and are extremely important in regulating all biological processes.

MicroRNA (miRNA) is a type of non-coding single-stranded RNA molecule coded by endogenous genes that measure 19-25 nt in length and play important roles in regulating biological evolution. MiRNA can influence the expression of target genes by post-transcriptional regulation during the processes of tumor incidence and development, biological development, organogenesis, epigenetic regulation, virus resistance, and so on. MiRNA and target genes usually exist in a one-to-many or many-to-one “regulate or be regulated” relationship (28). To analyze the connection between hub genes and miRNAs, we obtained hub gene–related miRNAs from Starbase (http://starbase.sysu.edu.cn/), which can provide predictions from a total of seven prediction procedures (TargetScan, microT, miRmap, picTar, RNA22, PITA, and miRanda), and we chose the relationships between miRNAs and messenger RNAs (mRNAs) that could be found in at least two of the procedures. We then constructed mRNA–miRNA regulatory networks and visualized them using Cytoscape.

Transcription factors (TFs) can control gene expression by interacting with target genes. We examined the relationships between TFs and hub genes from the MIRNet network to contrast hub gene–TF networks and analyze hub genes’ regulatory reactions. The hub gene–TF networks were then visualized by Cytoscape.

### 2.7 Identification and correlation analysis of immune cell infiltration among different subtypes in OA patients

The immune microenvironment is an integrated system that encompasses immune cells, inflammatory cells, fibroblasts, the mesenchyme, and various cytokines and chemokines. The analysis of immune cell infiltration in samples could play an important role in disease research and treatment prognosis. Single-sample GSEA (ssGSEA) is an extension of the GSEA method. In this research, we used ssGSEA to calculate the concentrations of 28 kinds of immune cells (29), then visualized the immune cell composition by box plot. Differences in immune cell proportions were estimated by the Wilcoxon test, and *p*< 0.05 was seen as statistically significant. CIBERSORx is based on machine learning and could extend this algorithm framework to analyze gene-expression profiles specific to certain cell types without the cells’ physical dissociation. RNA sequencing data were used to estimate the immune cell abundance (30). We estimated the abundance of 22 kinds of immune cells in OA patients of different subtypes from the dataset with the CIBERSORTx algorithm and drew a heatmap of immune cell infiltration correlation using the Corrplot R package (31).

The quantification of immune activity levels in tumor samples and the reflection of stromal and immune gene signatures by ESTIMATE analysis is a gene expression-based algorithm. The difference in immune scores of patients was estimated using the “estimate” R package (32) to calculate the hub genes’ correlations with immune scores.

### 2.8 Animal experiments

We bought three-month-old male-specific pathogen–free Sprague–Dawley rats from the Shanghai Institute of Planned Parenthood Research–BK Laboratory Animals Co., Ltd. (Shanghai, China) and divided them into two groups (n = 6 each). All procedures and protocols used in this study were approved by the ethical committee of Xin Hua Hospital, which is affiliated with the Shanghai Jiao Tong University School of Medicine (approval no. XHEC-F-2022-014). The rats were treated according to the 3R principles and housed at a temperature of 22 ± 2°C, under a 12-h light/dark cycle and humidity of 40-70%. All rats were intraperitoneally injected with 3% sodium pentobarbital (0.1 mL/100 g; Sigma-Aldrich, USA). Additionally, in the OA group, we injected 0.1ml of MIA (30 mg/mL; Aladdin Biochemical Technology Co., Shanghai, China) in the right knee joint space, whereas the control group received an equivalent volume of normal saline 0.9%. Each rat was reared for 4 weeks; then, we extracted cartilage tissues and synovial tissues after euthanasia. Next, the genes in the tissues were detected by qRT-PCR. As stated in the above results, MMP9, COL1A, and IGF1 were identified as high nodes interacting with 53, 47, and 4 genes, respectively. While MMP9 and FOS as hub genes interacted with 33 and 32 TFs, respectively. What is more? The PPI results showed that the differential genes may be enriched in the IL-17 pathway and other pathways. So, we chose MMP9, COL1A, IGF1, and IL-17 pathway-related proteins (IL-17A, Jak 2, JNK, MAPK 1, and STAT 3) to verify the expression of them. The primer sequences of each gene are shown in **Table 2**.

**Table 2:** The primer sequences.

Gene	Forward primer sequence	Reverse primer sequence
Gapdh	TCACTGCCACTCAGAAGACT	ACATTGGGGGTAGGAACACG
mmp9	GGTCCCCCTACTGCTGGTCCT	CGAGAACTTCCAATACCGACC
FOS	GGAGGACCTTATCTGTGCGT	TGCGGTTGCTTTTGATTTTT
COL1A	TATGTATCACCAGACGCAGAAGT	GCAAAGTTTCCTCCAAGACC
IGF1	ACGGGCATTGTGGATGAGTG	TGTGTCGATAGGGGCTGGGA
JNK	GGAGGAGCGAACTAAGAATGG	ACTGCTGTCTGTATCCGAGGC
JAK2	CCCTGGCTGTCTATAACTCC	TCTGTACCTTATCCGCTTCC
stat3	TTAACATTCTGGGCACGAAC	TCAGTGACAATCAAGGAGGC
IL-17A	CTACCTCAACCGTTCCACTT	ACTTCTCAGGCTCCCTCTTC
MAPK1	GGGCAGTTCTGGTCGTAGTGG	GGAAGGATTCAGGGCAGGGA

### 2.9 Statistical analysis

Data processing and analysis were completed in the R statistical language (version 4.1.1; R Foundation for Statistical Computing, Vienna, Austria). Continuous variables were compared between two groups by independent *t*-test to estimate normally distributed variables’ statistical significance, while two separate sets of variables were compared by Wilcoxon rank-sum test to estimate non-normally distributed variables’ statistical significance. Pearson correlation was used to calculate different genes’ correlation coefficients. The partial ROC (pROC) R package (33) was used for ROC curve analysis, and the area under the ROC curve (AUC) calculation was performed to evaluate the diagnostic model’s accuracy. All two-sided *p* values< 0.05 were considered statistically significant.

## 3 Results

### 3.1 Expression of OA-related genes in OA patients

**Figure 1:**
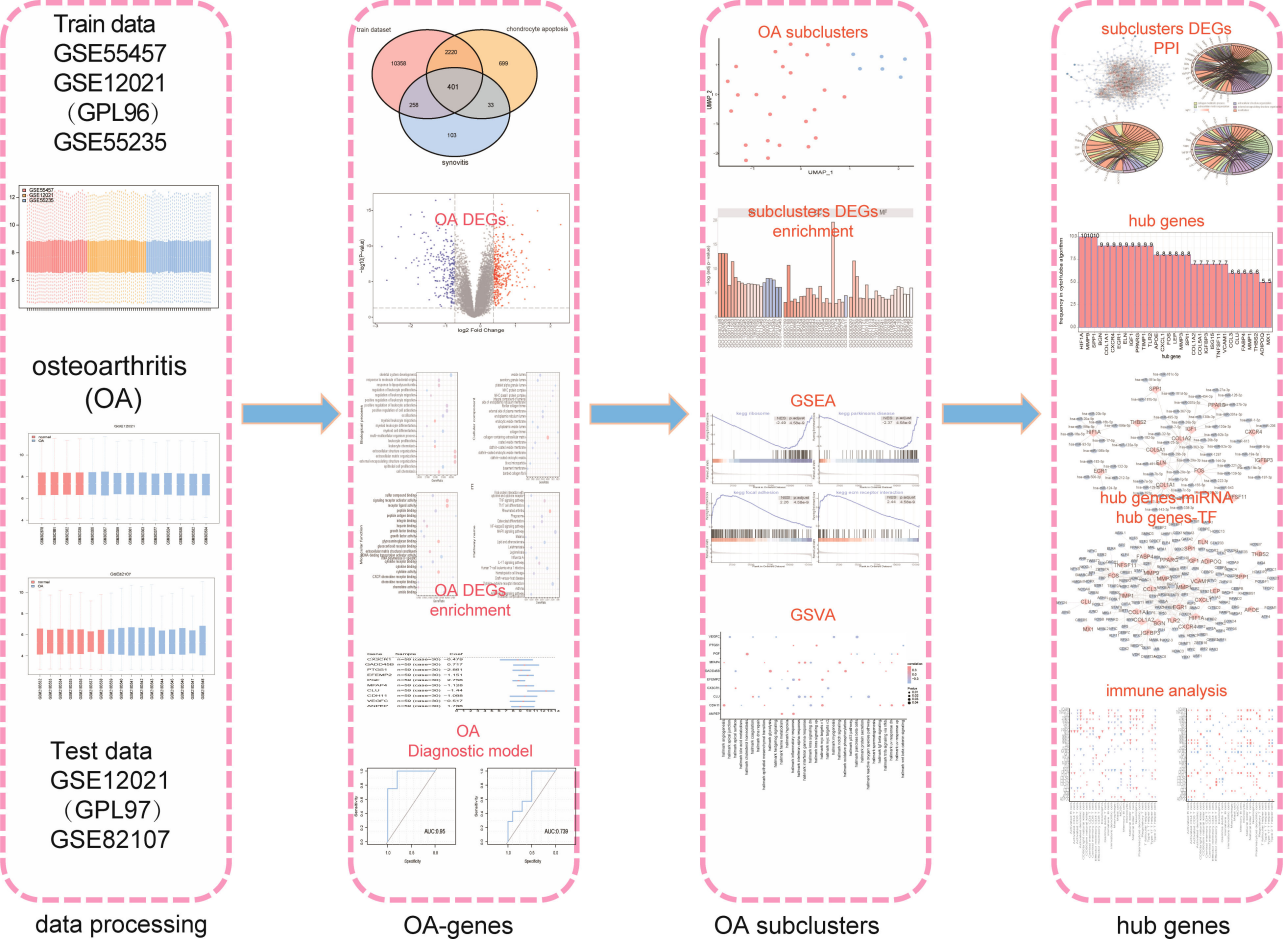
The flow chart.

**Figure 2:**
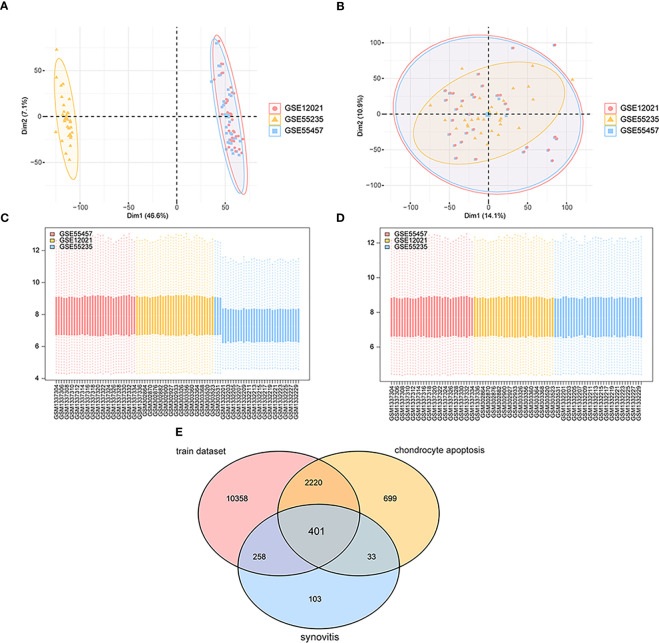
Data sets integration. **(A, C)** The maps of gene expression levels of integrated samples; the horizontal axis is the samples, and the vertical axis is the gene expression levels. **(B, D)** The maps of gene expression levels of integrated samples after the batch effects were removed; the horizontal axis is the samples, and the vertical axis is the gene expression levels. **(E)** The Venn map of OA-related genes; pink represents gene expression data in the training set, yellow means chondrocyte apoptosis-related genes, and blue means synovitis-related genes.

As shown in the flow chart (**Figure 1**), we first merged three datasets—GSE55457, GSE12021 (GPL96), and GSE55235—into a consolidated data set then removed significant batch effects (**Figures 2A, C**) between two groups of data to obtain gene-expression profiling data with consistent expression levels (**Figures 2B, D**). The consolidated data included 30 OA samples and 29 control samples. To screen OA-related genes, we searched keywords “synovitis” and “chondrocyte apoptosis” and found 795 synovitis-related genes and 3,353 chondrocyte apoptosis-related genes in GeneCards (14) then took the intersection with the consolidated gene-expression profiling data and obtained 401 OA-related genes (**Figure 2E**).

**Figure 3:**
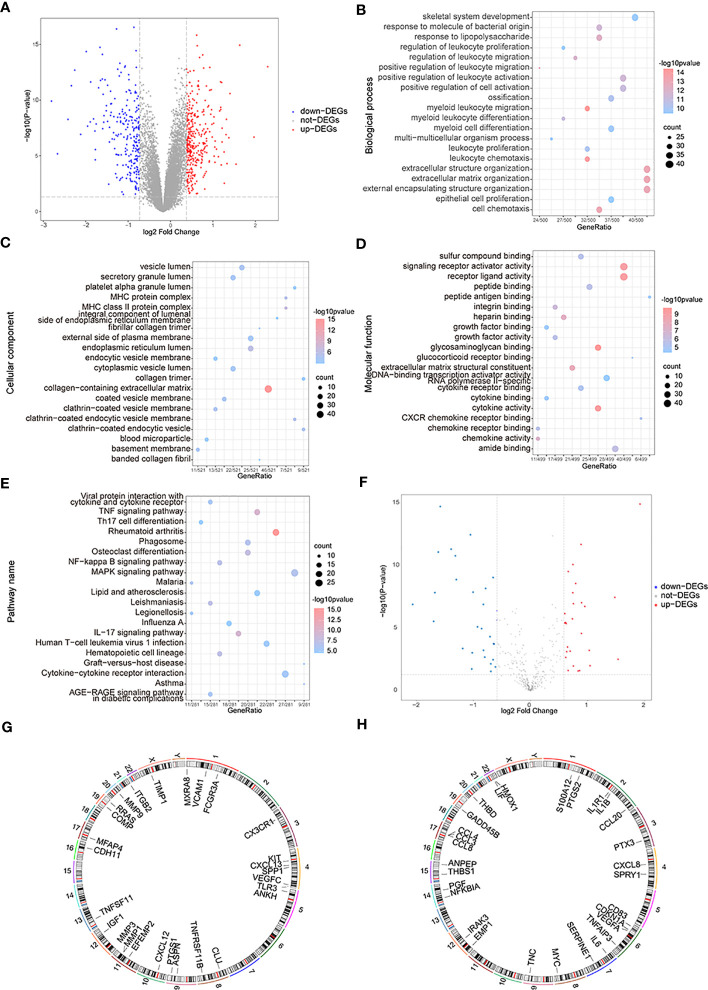
The functional enrichment analysis of differentially expressed genes (DEGs). **(A)** The volcano map of DEGs; the horizontal axis is log2FoldChange and the vertical axis is -log10(P-value); red nodes represent the up-regulated DEGs, blue nodes represent the down-regulated differentially expressed genes, and grey nodes mean the genes with no significant differences in expression level. **(B–E)** The BP, CC, MF, and KEGG analysis in GO terms of DEGs; the horizontal axis is gene ratio, the vertical axis is GO terms, the node sizes mean the genes’ numbers under each GEO term, and the color of the nodes means the significance level. **(F)** The volcano map of differentially expressed OA-related genes; the horizontal axis is log2FoldChange and the vertical axis is -log10(P-value); red nodes represent the up-regulated differentially expressed genes, blue nodes represent the down-regulated differentially expressed genes, and grey nodes means the genes with no significant differences in expression level. **(G, H)** The chromosome annotation of up or down-regulated differentially expressed genes. (KEGG, Kyoto Encyclopedia of Genes and Genome; GO, the Gene Ontology; BP, biological process; CC, cellular component; MF, molecular function).

The variance analysis between OA samples and control samples obtained 577 differential genes, which included 338 up-regulated genes and 239 down-regulated genes (**Figure 3A**). To analyze the two groups’ functional differences, we assessed the impacts of DEGs on the related biological functions of patients. For functional annotation of DEGs, GO enrichment analysis showed highly significant enrichment in the “myeloid leukocyte migration”, “leukocyte chemotaxis”, and “extracellular matrix” biological processes (**Figure 3B**); in the “collagen-containing extracellular matrix” and “endoplasmic reticulum lumen MHC protein complex” cellular components (**Figure 3C**); and in the “glycosaminoglycan binding”, “cytokine activity”, “receptor ligand activity”, and “signaling receptor activator activity” molecular functions (**Figure 3D**). These genes were also enriched in “rheumatoid arthritis”, “tumor necrosis factor (TNF) signaling pathway”, “IL-17 signaling pathway,” and “osteoclast differentiation” pathways in KEGG (**Figure 3E**). Taking the intersection of DEGs and OA-related genes, they yielded 56 differentially expressed OA-related genes (**Figure 3F**), including 27 up-regulated genes and 29 down-regulated genes. The RCircos R package was used to annotate up- or down-regulated genes on chromosomes (34) and showed that these genes appeared in a similar position (**Figures 3G, H**).

### 3.2 Risk model construction

**Figure 4:**
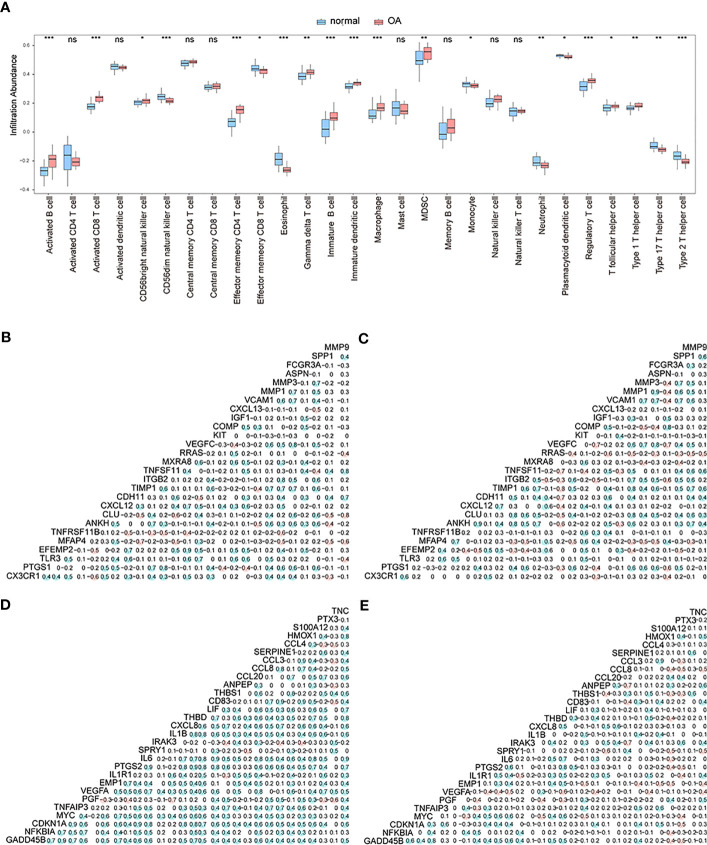
The correlation analysis. **(A)** The different enrichment levels of immune cells between OA samples and control samples; the horizontal axis is the immune cell, and the vertical axis is the enrichment level; *means the significant p-value was less than 0.05, **means the p-value was less than 0.01, and ***means the p-value was less than 0.001. **(B–C)** Correlation analysis of gene expression levels of up-regulated differentially expressed OA-related genes in both the control group and OA group; blue means positive correlation and red means negative correlation. **(D–E)** Correlation analysis of gene expression levels of down-regulated differentially expressed OA-related genes in both the control group and OA group; blue means positive correlation and red means negative correlation.

At this point, we performed ssGSEA to measure per-sample immune cell infiltration levels of control and OA groups, and the results showed that multiple immune cells’ infiltration levels were different between these two groups (*p*< 0.05) (**Figure 4A**). Specifically, the concentrations of gamma delta T-cells, immature B-cells, immature dendritic cells, and macrophages of OA samples were higher than those of the control samples.

We then analyzed the correlation in expression levels of 27 up-regulated genes and 29 down-regulated genes among the OA group and control group. The results showed that in the normal group, the expression levels of up-regulated (**Figure 4B**) and down-regulated (**Figure 4D**) genes were mostly positively correlated (p<0.05). In the OA sample group, the expression levels of up-regulated (**Figure 4C**) and down-regulated (**Figure 4E**) genes were mostly negatively correlated (p<0.05).

**Figure 5:**
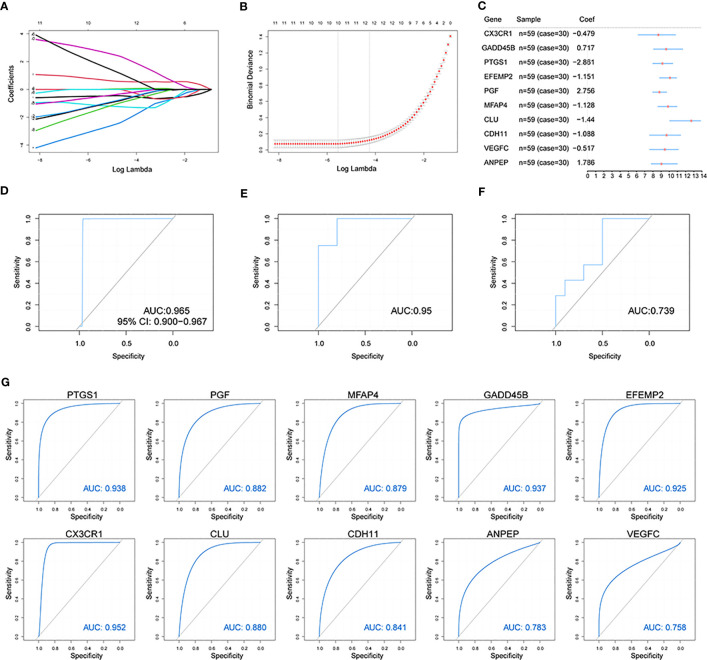
Construction of the osteoarthritis model. **(A, B)** The LASSO analysis was used to identify the characteristic genes. **(C)** The characteristic genes’ forest map of OA patients. **(D)** The ROC curve of predicted risk scores was used on the OA training set. **(E)** The ROC curve of predicted risk scores was used on the OA test set GSE12021(GPL97). **(F)** The ROC curve of predicted risk scores was used on the OA test set GSE82107. **(G)** The ROC curve of the 10 characteristic genes in OA diagnosis.

To estimate differentially expressed OA-related genes’ impact on OA patients, we used the LASSO algorithm to identify the following 10 characteristic genes with a great impact on OA among 56 differentially expressed OA-related genes: *CX3CR1*, *GADD45B*, *PTGS1*, *EFEMP2*, *PGF*, *MFAP4*, *CLU*, *CDH11*, *VEGFC*, and *ANPEP* (**Figures 5A, B**). An OA predictive risk score was estimated by multiplying and adding the 10 characteristic genes’ coefficients and gene-expression values. Each normalized expression value of the weighted penalty coefficient of characteristic genes was expressed by forest mapping (**Figure 5C**), and the predicted risk score of each sample was calculated to draw the ROC curve. The results included an AUC of 0.965 in the training set (**Figure 5D**). We then performed model validation involving the independent test data sets GSE12021(GPL97) and GSE82107, and the AUCs were 0.95 and 0.736 (**Figures 5E, F**), which indicated that the model prediction is good for OA patients. Similarly, the 10 characteristic genes were analyzed to predict OA ROC curves separately, and the results showed that all these genes had good predictive efficacy (**Figure 5G**).

**Figure 6:**
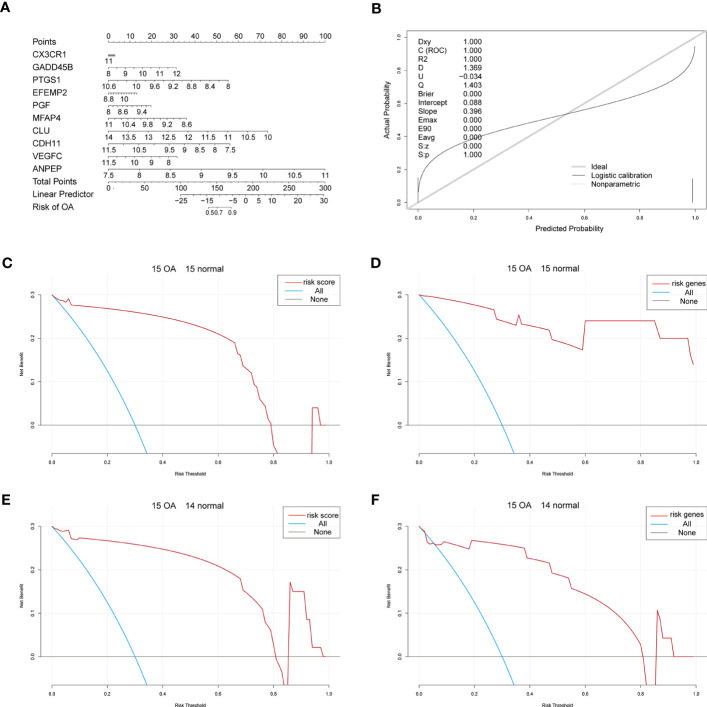
The nomogram. **(A)** The nomogram of the 10 characteristic genes in OA diagnosis. **(B)** The nomogram-corrected curve. **(C)** The DCA curve of predicted risk scores in the first group; blue means immediate diagnosis and pink represents the score risk model. **(D)** The DCA curve of the characteristic genes in the first group; blue means immediate diagnosis and pink means the combination of the characteristic genes. **(E)** The DCA curve of predicted risk scores in the second group; blue means immediate diagnosis and pink represents the score risk model. **(F)** The DCA curve of the characteristic genes in the second group; blue means immediate diagnosis and pink means the combination of the characteristic genes.

Considering patients’ predicted risk scores and the 10 characteristic genes, we built a nomogram model to predict OA patients’ prevalence rates and correct the nomogram model (**Figures 6A, B**). To assess the predictive model’s accuracy and predict the net benefits of patients who received intervention according to the model, we divided both OA samples and control samples into two groups, where the first group contained 15 OA samples and 15 control samples and the second group contained 15 OA samples and 14 control samples. The ggDCA R package (35) was used for decision curve analysis, and the predicted lines lying above the standard line indicate that the decision of the nomogram model might be beneficial for OA diagnosis (**Figures 6C–F**).

### 3.3 Identifying different OA subtypes according to characteristic genes

**Figure 7:**
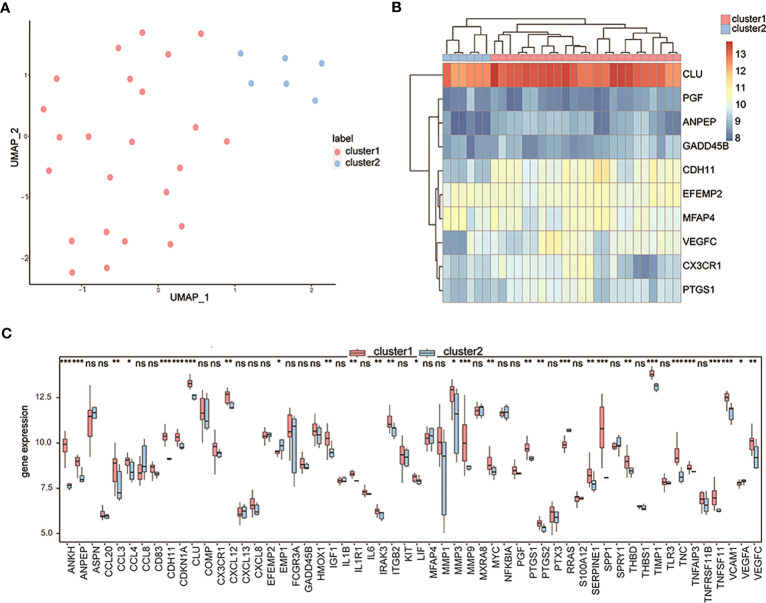
Clustering OA patients by the characteristic genes. **(A)** The UMAP clustering results; pink means cluster 1 and blue means cluster 2. **(B)** The expression heatmap of characteristic genes in two clusters; pink means cluster 1 and blue means cluster 2. **(C)** The expression differences of differentially expressed OA-related genes between cluster 1 and cluster 2; the horizontal axis is the characteristic gene, and the vertical axis is the gene expression level. ns means P≥0.05 with no statistical significance; * means P<0.05; ** means P<0.01; *** means P<0.001.

Considering the 10 OA-related genes, a pair of OA subtypes, cluster 1 and cluster 2, were identified by the UMAP algorithm (**Figure 7A**), with 24 samples in cluster 1 and 6 samples in cluster 2. The clustering results showed significant differences in characteristic genes between the two groups (**Figure 7B**). The expression levels of differentially expressed OA-related genes of both subtypes in the control and OA groups were measured, and the results showed that most differentially expressed OA-related genes in the two groups were also differentially expressed in both subtypes (**Figure 7C**).

### 3.4 Enrichment analysis and network analysis

**Figure 8:**
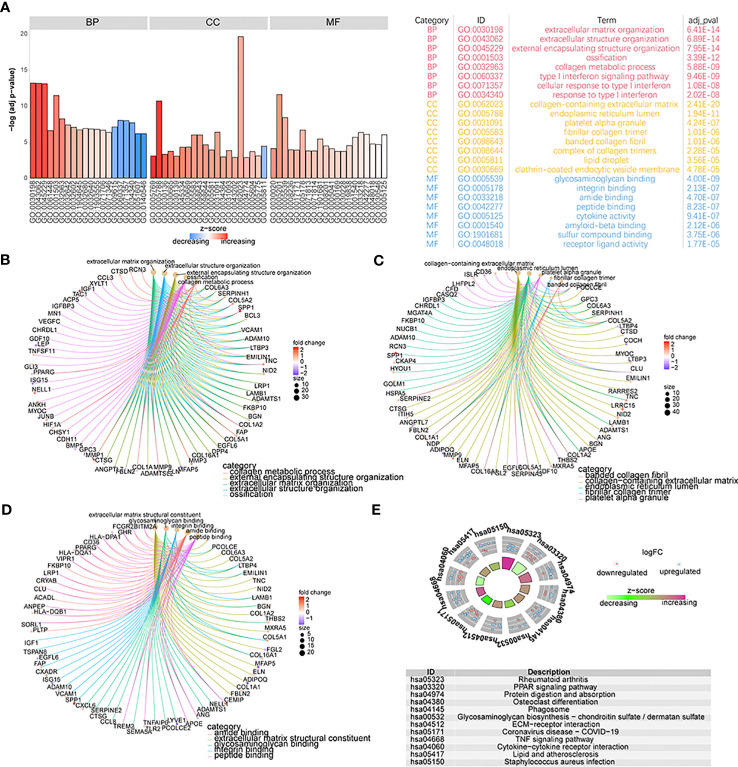
The functional analysis of DEGs. **(A)** GO functional enrichment analysis of DEGs; the horizontal axis is GO terms and the vertical axis is the significance of enrichment results. **(B–D)** The results of the first five items of BP, CC, and MF analysis; the node size means the genes’ number which was enriched under each term; different line colors mean different biological functions. **(E)** The KEGG enrichment analysis; different node colors mean different gene expression levels and the quadrilateral color means the Z-score of KEGG pathways.

To detect the biological differences between patients with the two different OA subtypes, we first obtained 355 DEGs by analyzing both groups of patients’ gene-expression profiles. We performed GO annotation of these DEGs and found these genes are involved in many biological processes (**Figure 8A**; **Supplementary Table 2**-go). Specifically, the results showed that these genes were mainly enriched in biological processes such as extracellular matrix organization, extracellular structure organization, extracellular encapsulating structure organization, and ossification (**Figure 8B**); cellular components such as collagen-containing extracellular matrix, endoplasmic reticulum lumen, platelet alpha granule, and fibrillar collagen trimer (**Figure 8C**); and molecular functions such as extracellular matrix structural constituent, glycosaminoglycan binding, integrin binding, and amide binding (**Figure 8D**). Enrichment in KEGG pathways such as rheumatoid arthritis, PPAR signaling pathway, protein digestion and absorption, and osteoclast differentiation was also noted (**Figure 8E**; **Supplementary Table 2**-kegg).

**Figure 9:**
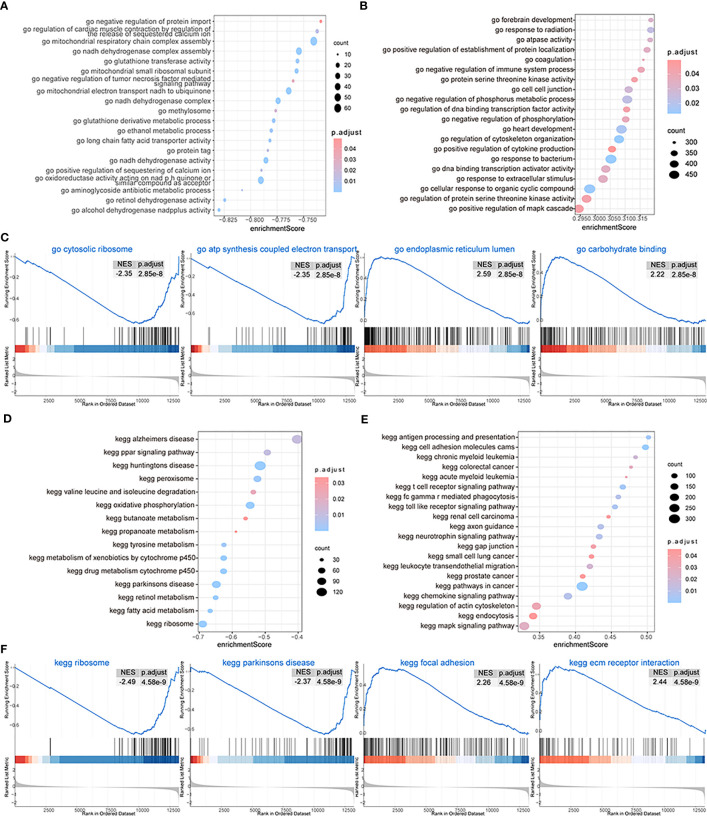
GSEA. **(A, B)** The GSEA-GO analysis; the biological process is inhibited **(A)** and the biological process is activated **(B)** in cluster 1; the horizontal axis is the enrichment score, and the vertical axis is GO terms. The color means the p-value and the node size means the enriched genes’ number. **(C)** The first four items of GO terms. **(D, E)** The GSEA-KEGG analysis; the biological process is inhibited **(D)** and the biological process is activated **(E)** in cluster 1; the horizontal axis is the enrichment score, and the vertical axis is KEGG terms. The color means the p-value and the node size means the enriched genes’ number. **(F)** The first four items of KEGG terms.

We then performed GSEA considering both subtypes of OA patients and found that biological processes such as GO structural constituents of ribosomes, GO oxidoreductase activity acting on NAD pH quinone or a similar component as an acceptor, GO mitochondrial respiratory chain complex assembly, GO ribosomal subunit, and GO ATP synthesis-coupled electron transport could be inhibited in patients from cluster 1 (**Figure 9A**), while biological processes such as GO endoplasmic reticulum lumen, GO collagen fibril organization, GO endoderm formation, and GO neuroinflammatory response were promoted (**Figures 9B, C**; **Supplementary Table 3**-gsea-go). The pathway activity of patients from the two subtype groups was analyzed, and the results showed that pathways such as the ribosome, Parkinson’s disease, drug metabolism cytochrome p450, and metabolism of xenobiotics by cytochrome p450 were inhibited in cluster 1 patients (**Figure 9D**), while pathways such as ECM receptor interaction, lysosome, focal adhesion, and *Leishmania* infection were promoted (**Figures 9E, F**; **Supplementary Table 3**-gsea-kegg).

**Figure 10:**
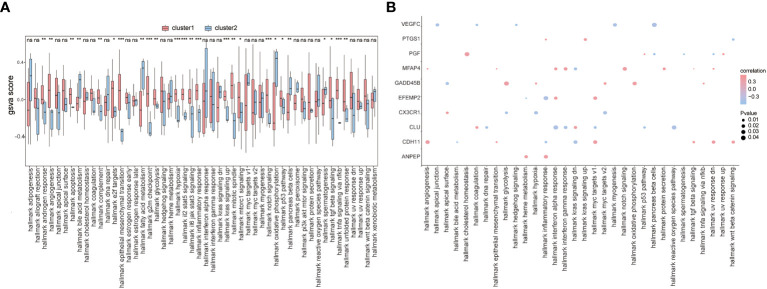
GSVA. **(A)** The difference in hallmark between the two subtypes of patients; the horizontal axis is the hallmark, and the vertical axis is GSVA scores; pink means cluster 1 and blue means cluster 2, *means the significant p-value was less than 0.05, **means the p-value was less than 0.01, and ***means the p-value was less than 0.001. **(B)** The correlation of characteristic genes and hallmark; the horizontal axis is hallmark, and the vertical axis is characteristic genes; the node size means the significance level and the node color means the correlation level.

To further explore the functional differences between the two subtypes, we used GSVA and found that biological processes such as hallmark hypoxia, hallmark interleukin-2 STAT5 signaling, hallmark interleukin-6 JAK/STAT3 signaling, and hallmark inflammatory response were significantly activated in cluster 1 patients (**Figure 10A**). Concurrently, most of the other biological processes, such as hallmark notch signaling, hallmark oxidative phosphorylation, hallmark p53 pathway, and hallmark pancreas beta cells, showed significant differences between the two groups of patients (**Figure 10A**). We also analyzed the correlation between patients’ characteristic genes and hallmark biological processes, and the results showed that MFAP4 and hallmark TGF beta signaling, hallmark epithelial-mesenchymal transition, or hallmark angiogenesis were significantly positively correlated (*p*< 0.05), while EFEMP2 and hallmark heme metabolism, PGF and hallmark spermatogenesis, hallmark UV response dn, and hallmark pancreas beta cells were significantly negatively correlated (*p*< 0.05) (**Figure 10B**).

### 3.5 Network analysis between two subtypes of patients

**Figure 11:**
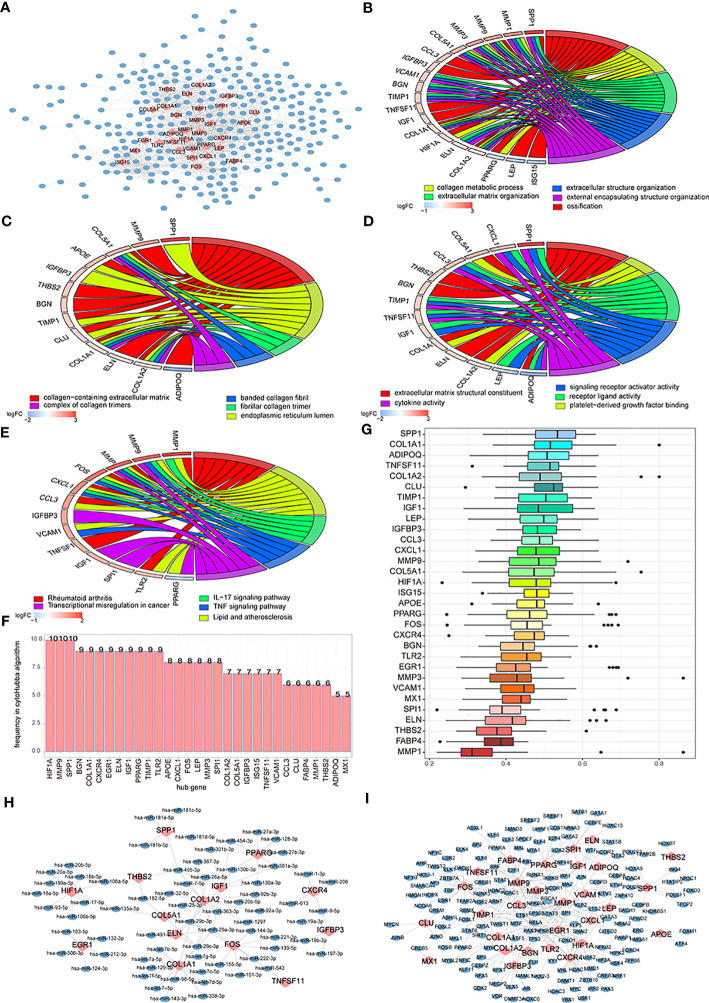
The differentially expressed genes (DEGs)-associated networks. **(A)** DEGs’ protein-protein interaction (PPI) network; the blue node represents DEG, and the pink node means hub genes. **(B–E)** The BP, CC, MF, and KEGG analysis of genes’ GO terms in the PPI network; the node color means genes’ expression level, and the different line color means different biological functions. **(F)** The genes frequency tables of 12 algorithms; the horizontal axis is genes, and the vertical axis is frequency. **(G)** The GO semantic similarity scores of hub genes in DEGs’ PPI network; the horizontal axis is the similarity level, and the vertical axis is the gene. **(H)** Hub genes’ mRNA-miRNA network; the pink node means hub genes and the blue node means miRNA. **(I)** Hub genes’ mRNA-TF network; the pink node means hub genes and the blue node means TF.

To analyze the impact of the two subtypes of patients’ DEGs on osteoarthritis patients’ biological functions, we first built subtypes of patients’ DEGs-related PPI networks and visualized the results using Cytoscape. The PPI networks contained 451 interaction pairs and 349 DEGs, with an average node degree of 2.58, an average local clustering coefficient of 0.404, and a PPI enrichment *p*-value< 1.0 (15). Among them, mmp9, COL1A, and IGF1 were high-degree nodes that interacted with 53, 47, and 4 genes, respectively (**Figure 11A**). To analyze the effects of genes in the PPI network on osteoarthritis, we performed enrichment analysis involving genes from the network and determined that these genes mainly affected biological processes like ossification, collagen metabolic process, and extracellular matrix organization (**Figure 11B**); cellular components like collagen-containing extracellular matrix, endoplasmic reticulum lumen, and fibrillar collagen trimer (**Figure 11C**); cell functions like extracellular matrix structural constituent, platelet-derived growth factor binding, and receptor-ligand activity (**Figure 11D**); and signaling pathways like rheumatoid arthritis, lipid and atherosclerosis, the A signaling pathway, and the TNF signaling pathway (**Figure 11E**).

We used 12 algorithms of CytoHubba to calculate the top 30 nodes in each algorithm and extracted 31 genes we called hub nodes from at least five algorithms (**Figure 11F**). Then, the GOSemSim R package was used to analyze the hub genes’ GO semantic similarity (36), and the results showed that RPL19, RPS11, and RPL10A had greater functional correlations with multiple genes (**Figure 11G**).

We built a hub gene mRNA–miRNA network. The network contained 97 interactions, which included 14 mRNAs and 68 miRNAs, in which COL1A1 and COL1A2 hub genes could both interact with 14 miRNAs (**Figure 11H**). The hub genes’ mRNA–TF network was also built and contained 29 miRNAs and 167 TFs; among these, hub genes mmp9 and Fos could interact with 33 and 32 miRNAs, respectively (**Figure 11I**).

### 3.6 Differences in immune characteristics between RNA modification patterns

**Figure 12:**
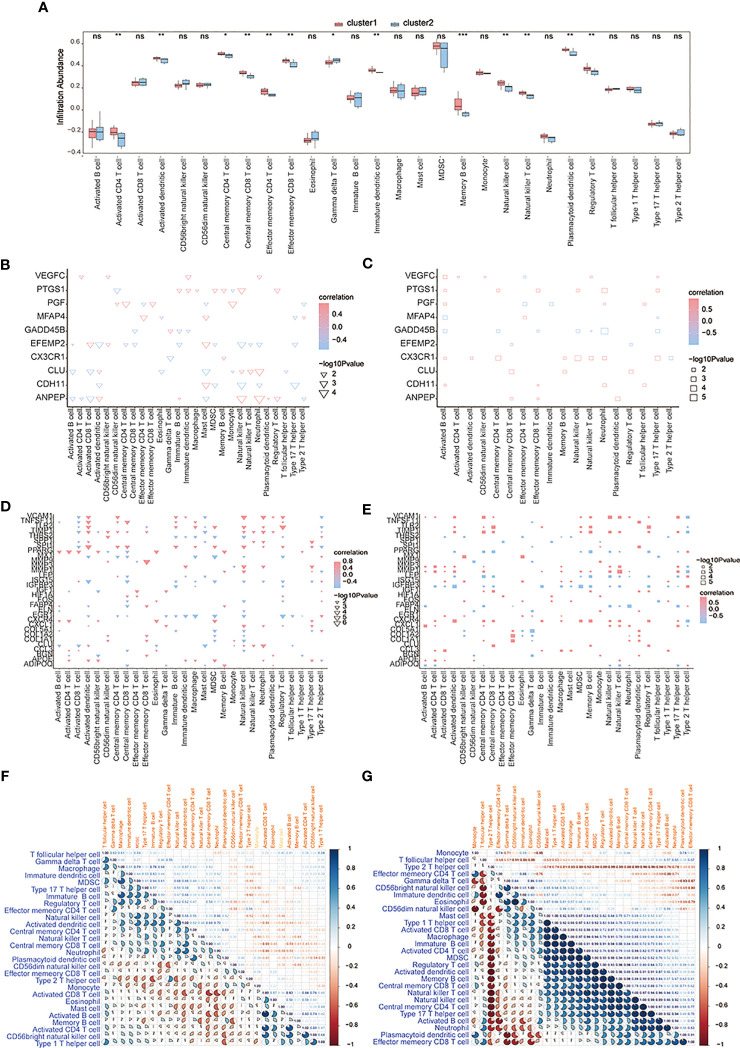
The immune characteristics-ssGSEA between two subtypes of OA patients. **(A)** The content histogram of immune cells between cluster 1 and cluster 2 patients; the blue means cluster 2 sample while the pink one means cluster 1 sample; the horizontal axis is the immune cell, and the vertical axis is cell content. **(B, C)** The correlation of characteristic genes and immune cells between cluster 1 and cluster 2; the node size means significance and the node color means correlation; the horizontal axis is the immune cell and the vertical axis is characteristic genes. **(D, E)** The correlation of hub genes and immune cells between cluster 1 and cluster 2; the node size means significance and the node color means correlation; the horizontal axis is the immune cell, and the vertical axis is the hub gene. **(F, G)** The correlation analysis of immune cells in cluster 1 and cluster 2; red means negative correlation while blue means positive correlation. ns means P≥0.05, with no statistical significance; * means P<0.05; ** means P<0.01; *** means P<0.001.

CIBERSORTx and ssGSEA were used to compare immune cell infiltration levels between osteoarthritis patients of two subtypes. ssGSEA showed that patients’ concentrations of central memory CD4^+^ T-cells, central memory CD8^+^ T-cells, effector memory CD4^+^ T-cells, effector memory CD8^+^ T-cells, natural killer cells, and natural killer T-cells in cluster 1 were significantly higher than those in cluster 2 (**Figure 12A**). We computed the correlation of characteristic genes and immune cells between cluster 1 and cluster 2 patients, and the results indicated that activated CD8^+^ T-cells and activated dendritic cells were significantly correlated with multiple characteristic genes’ expression levels (*p*< 0.05) (**Figure 12B**) in cluster 1, while in cluster2, activated B-cells were significantly related to the characteristic genes’ expression levels (*p*< 0.05) (**Figure 12C**). The correlations of hub genes and immune cells in cluster 1 and cluster 2 were respectively calculated, and we found that hub gene *PPARG* showed a stronger correlation with multiple immune cells in cluster 1 (*p*< 0.05) (**Figure 12D**), while hub genes *MMP1* and *MMP3* were highly related to multiple immune cells in cluster 2 (*p*< 0.05) (**Figure 12E**). We also estimated the correlation among various immune cells of both groups of patients and found that the correlations were weak in cluster 1 (**Figure 12F**), while in cluster 2, type 1 T helper cells, activated CD8^+^ T-cells, macrophages, immature B-cells, activated CD4^+^ T-cells, MDSCs, regulatory T-cells, activated dendritic cells, memory B-cells, central memory CD8^+^ T-cells, natural killer T-cells, natural killer cells, central memory CD4^+^ T-cells, type 17 T helper cells, and activated B-cells were positively correlated; however, there were negative correlations among T follicular helper cells, type 2 T helper cells, and most other immune cells (**Figure 12G**).

**Figure 13:**
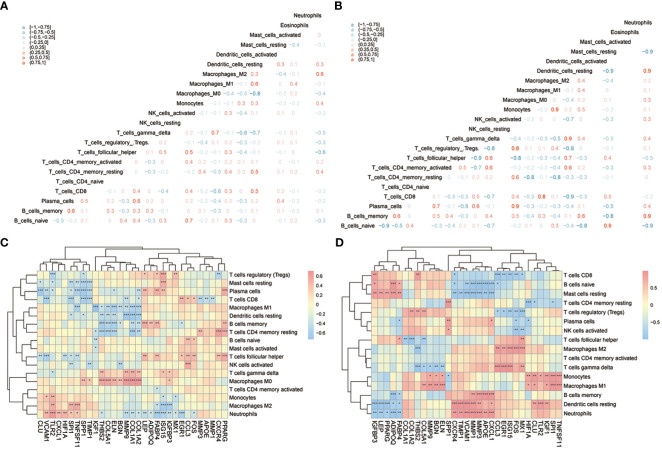
The immune characteristics-CIBERSORTX between two subtypes of OA patients. **(A, B)** The correlation of immune cells’ content between cluster 1 and cluster 2 patients; pink means positive correlation while blue means negative correlation. **(C, D)** The correlation of immune cells and hub genes between cluster 1 and cluster 2 patients; the horizontal axis is the hub gene, and the vertical axis is the immune cell; red means positive correlation while blue means negative correlation. The node size means significance and the node color means correlation. *means the significant p-value was less than 0.05, **means the p-value was less than 0.01, and ***means the p-value was less than 0.001.

Next, CIBERSORTx was used to compare immune cell infiltration levels between the two subtypes of patients, and the results showed that the correlation of various immune cell concentrations between cluster 1 and cluster 2 was significantly different (*p*< 0.05) (**Figures 13A, B**). We then calculated the correlations of 31 hub genes and immune cell contents separately and found that M1 macrophages and dendritic cells were significantly negatively correlated with multiple hub genes (**Figure 13C**), while gamma delta T-cells and M0 macrophages were significantly positively correlated with the same genes (**Figure 13D**).

**Figure 14:**
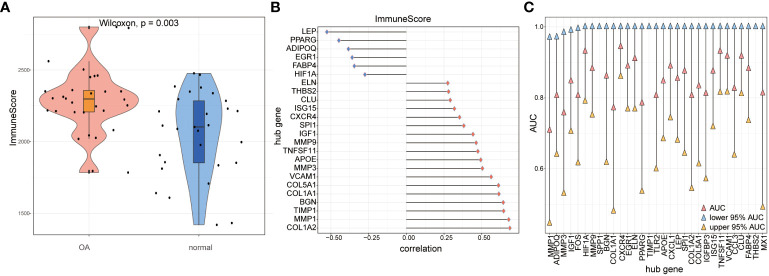
The immune score calculation. **(A)** The immune score of OA samples and control samples; red means OA samples while blue means control samples; the vertical axis is the immune score. **(B)** The correlation of immune score and hub genes in all the OA samples; the horizontal axis is the correlation, and the vertical axis is hub genes. **(C)** AUC and 95% AUC in the ROC curve of hub genes, the blue node means lower 95% AUC, yellow means upper 95% AUC, pink means AUC, the horizontal axis is hub genes, and the vertical axis is AUC values.

We compared immune scores between OA samples and control samples and found that the OA samples’ scores were significantly higher than those of the control samples (*p*< 0.05) (**Figure 14A**). Then, the correlations of hub genes’ expression levels and immune scores were calculated, and the results showed that hub genes such as *FABP4*, *EGR1*, *ADIPOQ*, *PPARG*, and *LEP* were negatively correlated with immune scores, while hub genes such as COL1A2, *MMP1*, *TIMP1*, *BGN*, and COL1A1 were positively correlated with them (*p*< 0.05) (**Figure 14B**). To estimate the ability to distinguish the two subtypes of OA according to hub genes, we computed the AUC score using the ROC curve and found that genes such as *TNFSF11*, *VCAM1*, *CCL3*, *CLU*, *FABP4*, and *THBS2* could distinguish between the two subtypes very well (**Figure 14C**).

### 3.7 RT-qPCR validation results

**Figure 15:**
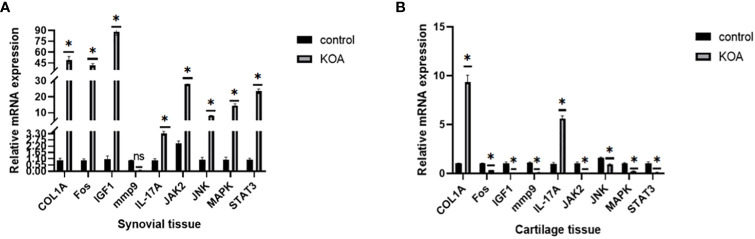
PCR results. **(A)** Expression levels of related proteins in cartilage tissues. **(B)** Expression levels of related proteins in synovial tissues (ns: p>0.05, *: p<0.05).

As mentioned above, we used 2-month-old SD rats for the following studies. After KOA modeling, the same batch of rats were randomly selected for knee joint staining to verify the success of KOA model (**Supplementary Figure 1**). After KOA modeling, cartilage tissue, and synovial tissue were collected from two groups of rats for PCR verification. The expression levels of the COL1A, Fos, IGF1, mmp9, IL-17A, Jak2, JNK, MAPK1, and STAT3 nodes were verified in PCR rats’ tissues, and the results are shown in **Figure 15**. All the genes tested were different in the OA group, but this trend was not completely consistent. Only COL1A and IL-17A were highly expressed in cartilage and synovium, which is consistent with the bioinformatics prediction.

## 4 Discussion

Osteoarthritis (OA) is the most common joint disease and shows an increased incidence with age (37). An imbalance in the catabolism and anabolism of cartilage (38) and pain-related synovitis (39) could affect the development of OA, and synovium might induce an early response in OA by regulating cartilage development and proteolysis (40). Thus, both synovium and cartilage are important in OA progression and could underly the therapeutic potential in OA. Synovial inflammation can induce articular cartilage injury, while cartilage injury can further aggravate synovial inflammation (41). To date, however, no well-defined target or treatment mechanism exists for either phenotype, so we performed a bioinformatics analysis on the results of microarray and high-throughput technology, identified and validated *in vivo* the DEGs associated with both synovitis and cartilage apoptosis, then analyzed immune cell infiltration and subtype classification for an in-depth understanding of the mechanisms of OA.

The present study considered 577 differently expressed genes and 401 synovitis or chondrocyte apoptosis-related genes whose intersection revealed 56 differential expressed OA-related genes. Several biological processes, cellular components, and molecular functions were enriched categories in the GO analysis of DEGs, while KEGG analysis revealed the DEGs were involved in the TNF signaling pathway, IL-17 signaling pathway, and other pathways. TNF-α transmits signals through TNF receptor 1 (TNF1) and TNF receptor 2 (TNF2) in the TNF pathway (42), and TNF-α can also be released by adipose tissue to negatively regulate by promoting matrix metalloproteinase generation and inhibiting proteoglycans or type II collagen synthesis (43). Intra-articular injection of IL-17-neutralizing antibodies could decrease the expression of joint-degeneration markers (44), and a holistic study showed that hub genes in OA were significantly enriched in the IL-17 signaling pathway (45). These conclusions are consistent with the results of our analysis and the fact that pathway protein interleukin-1A was highly expressed in the synovium and cartilage of OA rats.

The levels of multiple immune cells in OA samples, such as gamma delta T-cells, immune B-cells, immature dendritic cells, and macrophages, were higher than those in the control group according to ssGSEA, and 10 characteristic genes were identified from 56 differential expressed OA-related genes by LASSO algorithms, i.e., *CX3CR1*, *GADD45B*, *PTGS1*, *EFEMP2*, *PGF*, *MFAP4*, *CLU*, *CDH11*, *VEGFC*, and *ANPEP*. We then calculated predictive risk scores and used ROC curves to obtain results showing that these genes have good predictive abilities; moreover, the nomogram model decisions, which were made based on the predicting risk scores and 10 characteristic genes, might be beneficial to OA diagnosis.

We then used UMAP methods to divide OA patients into cluster 1 and cluster 2. Notably, most of the genes differentially expressed between OA and control samples were also differentially expressed between these two clusters, such as *IGF1*, *MMP9*, and *CX3CR1*. Also, a PCR experiment in rats’ tissues showed that the level of IGF1 in OA rats’ synovium was higher than that in control rats, but the trend was exactly opposite in the cartilage, while the trends of MMP9 were the same in both tissues with low expression in OA samples and high expression in control samples, contrary to the bioinformatic analysis results. Insulin-like growth factor 1 (IGF-1) can promote longitudinal bone growth (46) and support chondrocyte survival, proliferation, or cartilage matrix synthesis *via* PI3K/AKT, MAPK, and NF-kB pathways (47, 48); however, whether its expression level will change with OA progression and tissue type and finally lead to the difference between rats and human patients and between cartilage and synovium needs to be elaborated. Still, the differentially expressed level of insulin-like growth factor 1 in the synovium of OA was first mentioned in this study and might be a new therapeutic target in synovitis in early-stage OA. Additionally, studies focusing on OA showed that it may be a potential diagnostic marker of OA given the higher levels recorded in OA cartilage tissue than in control cartilage tissue (49, 50) and with its leading role in the intima layer’s macrophages in early-stage OA synovitis (51). Our analysis concerning MMP9 also found an expression difference between OA and control samples, but the trends were totally different and remain to be further investigated by different experimental or modeling methods.

A total of 355 DEGs were identified from the expression profiles of the two OA subtypes and subsequently enriched using GO, KEGG, and GSEA. Then, functional differences between the two subtypes were analyzed using GSVA. Subsequently, we constructed a PPI network of DEGs among OA subtypes and identified three highly connected nodes: MMP9, COL1A, and IGF1. The results of the gene-enrichment analysis showed that these genes were mainly enriched in the IL-17 pathway, TNF pathway, and other signaling pathways. Both MMP9 and IGF1 have been discussed previously, while COL1A is often considered a marker of osteoblast differentiation (52), and the polymorphism of the transcription factor SP1 binding site is closely related to bone mass and fracture (53). Simultaneously, as a fibrosis mark, COL1A could also promote articular cartilage repair after injury (54). The PCR results in rat tissues showed that the expression level of COL1A in the OA group was higher than that in the control group both in cartilage tissues and synovial tissues, which indicated that the repair mechanisms in the cartilage of OA rats might be activated, while synovium might also undergo fibrosis. We also first looked at the effect of COL1A on synovitis in OA.

A total of 31 hub genes were found and the GOSemSim R package showed that *ADIPOQ*, *COL1A1*, and *SPP1* were closely related to the function of several genes. Adiponectin (*ADIPOQ*) is released from adipose tissue and plays an important role in bone formation and resorption (55); it is involved in the inflammatory response and triggers cartilage damage by up-regulating the expression of cytokines, matrix-degrading enzymes, and chemokines in chondrocytes and synovial fibroblasts (56). Phosphoprotein 1 (*SPP1*) is an extracellular matrix adhesion molecule that plays important roles in bone mineralization, immune response, tumor metastasis, inflammation, and angiogenesis (57), and it has also been identified to be a regulator of the PI3K/AKT pathway and could influence chondrocyte status in OA (58). These findings are consistent with the results of our analysis, but experimental verification of *ADIPOQ* and *SPP1* is lacking in the present study.

We constructed mRNA-miRNA and mRNA-TF networks of hub genes and found that *COL1A1* and *COL1A2* interacted with 14 miRNAs, respectively, while *MMP9* and *Fos* interacted with 33 and 32 TFs, respectively. During rat tissue’s PCR validation, the expression of *Fos* was increased in synovium but decreased in cartilage from the OA group compared to the control group. *C-fos* could form a heterodimeric *AP-1* complex with *C-Jun* (59). Previous studies have shown that *C-fos* could promote osteoclast fusion and accelerate osteoclastogenesis *via* the *ERK*/*C-Fos*/*NFATc1* pathway (60), and *C-Fos*/*AP-1* could also drive synovial mesenchymal stem cells to generate pannus, invade the cartilage and bone, and release interleukin-1β (61), which eventually activates downstream matrix metalloproteinase and induces cartilage destruction *via C-Fos*/*AP-1* (62). Therefore, the synovium of our OA rats might be activated by *C-Fos*, while the cartilage might be in the compensatory stage of repair after injury. The conclusion needs to be further verified.

Finally, we used ssGSEA and CIBERSORTx algorithms to compare the immune status between the two subtypes. The results showed that there were differences in the concentrations of immune cells, the correlation between characteristic genes and immune cells, the correlation between hub genes and immune cells, and the correlation between the content of immune cells between cluster 1 and cluster 2 patients. What is more? The results of ROC curve analysis and AUC scores showed that *TNFSF11*, *VCAM1*, *CCL3*, *CLU*, *FABP4*, and *THBS2* could effectively distinguish the two subtypes of OA. This analysis helps to further the understanding of the immune status contrast between OA and control samples and between the two subtypes of OA.

However, there were limitations in this study; for example, *in vivo* verification experiments were only performed on synovium and cartilage tissues of MIA-intervened OA rats, without the use of different modeling methods or other species. In addition, only qRT-PCR was used to verify the bioinformatics analysis results, and no other experiments, such as western blotting, immunofluorescence, or immunohistochemistry, were used to validate at the cell or tissues level, so the validation results are limited, while further validation of 10 key genes could also provide more information for our research on OA, which is also the shortcoming of the study.

In summary, the present study screened several genes and pathways closely related to synovitis and cartilage degradation in OA through bioinformatics analysis. Notable genes include *CX3CR1*, *GADD45B*, *PTGS1*, *EFEMP2*, *PGF*, *MFAP4*, *CLU*, *CDH11*, *VEGFC*, *ANPEP*, *MMP9*, *COL1A*, *Fos*, *IGF1*, *ADIPOQ*, and *SPP1*. Key pathways include the *IL-17* signaling pathway, TNF signaling pathway, and *p53* pathway. The expression levels of *MMP9*, *COL1A*, *Fos*, *IGF1*, and *IL-17* pathway-related proteins *IL-17A*, *ERK1*, *JAK2*, *JNK*, *MAPK1*, and *STAT3* were confirmed by RT-PCR in rats’ tissues, with *IL-17A* highly expressed in both synovium and cartilage of KOA rats and with lower expressions of MMP9 in both tissues; the former two findings are consistent with the prediction, while the latter finding is the exact opposite. These results suggest that chondrocyte repair or synovial fibrosis might exist in OA rats, and the *IL-17* pathway might also be activated in OA rats. The *IL-17A*, *COL1A*, and *MMP9* screening performed in this study might yield therapeutic targets for synovitis and cartilage apoptosis in OA.

## Data availability statement

The datasets presented in this study can be found in online repositories. The names of the repository/repositories and accession number(s) can be found in the article/**Supplementary Material**.

## Ethics statement

The animal study was reviewed and approved by Xin Hua Hospital, which is affiliated with Shanghai Jiao Tong University School of Medicine (Approval No. XHEC-F-2022-014).

## Author contributions

LY: Experimental operation, Data management and analysis; XY: Experimental operation, Writing manuscript, Writing-review and editing; ML: Supervision, Writing-review; YC: Conceptualization, methodology, supervision, Writing-review and editing. All authors contributed to the article and approved the submitted version.
