# Supplementary figures and images for: A comprehensive analysis of biomarkers associated with synovitis and chondrocyte apoptosis in osteoarthritis
Source: Front Immunol. 2023 Jul 21;14:1149686. doi: 10.3389/fimmu.2023.1149686 (PMC10401591; doi:10.3389/fimmu.2023.1149686)

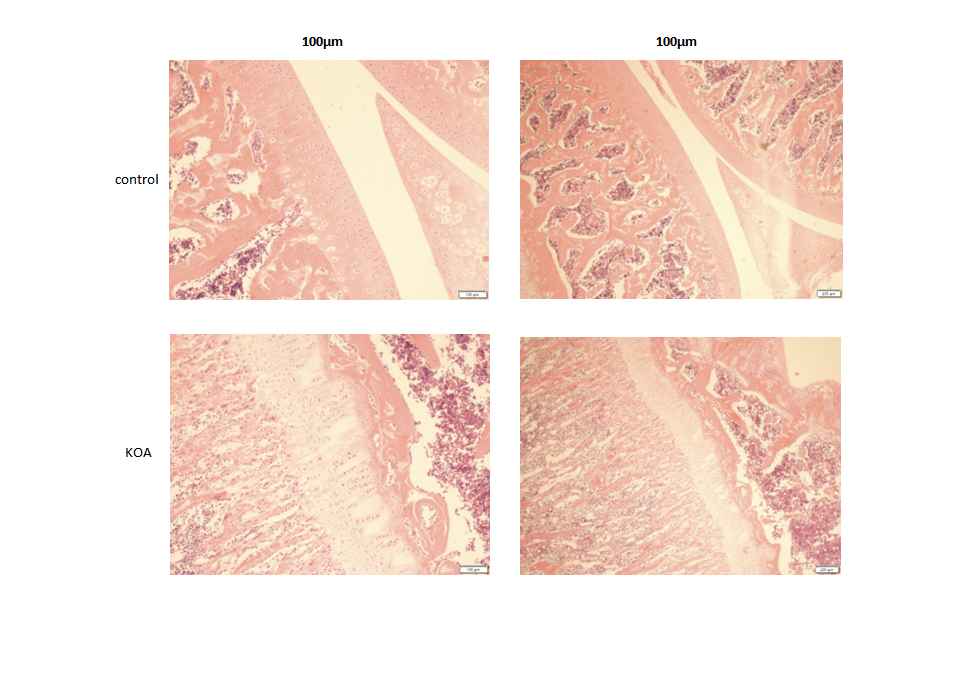

Supplement: Supplementary file 1 [file Image_1.png]
